# Changes in Ocular Surface Characteristics after Switching from Benzalkonium Chloride-Preserved Latanoprost to Preservative-Free Tafluprost or Benzalkonium Chloride-Preserved Tafluprost

**DOI:** 10.1155/2017/3540749

**Published:** 2017-08-02

**Authors:** Naoto Tokuda, Yasushi Kitaoka, Akiko Matsuzawa, Ayaka Tsukamoto, Kana Sase, Shinsuke Sakae, Hitoshi Takagi

**Affiliations:** Department of Ophthalmology, St Marianna University School of Medicine, Kanagawa, Japan

## Abstract

**Purpose:**

The aim of the present study was to examine the effects of switching from Latanoprost ophthalmic solution containing a preservative to preservative-free Tafluprost ophthalmic solution or Tafluprost containing a preservative on ocular surfaces.

**Materials and Methods:**

Forty patients (40 eyes) with glaucoma (mean age: 62.0 ± 10.9 years) using Latanoprost with preservative for six months or longer were assigned either to a Tafluprost-containing-preservative group (20 eyes) or preservative-free-Tafluprost group (20 eyes). The intraocular pressure, corneal epithelial barrier function (fluorescein uptake concentration with fluorophotometer FL-500), superficial punctate keratopathy (AD classification), and tear film breakup time (TBUT) were assessed before switching and at 12 weeks after switching.

**Results:**

No significant differences in intraocular pressure were noted after switching in either group. Corneal epithelial barrier function was improved significantly after switching in both the Tafluprost-containing-preservative and the preservative-free-Tafluprost groups. There were no significant differences in AD scores after switching in the Tafluprost-containing-preservative group, but significant improvements were noted in the preservative-free-Tafluprost group. No significant differences in TBUT were noted in the Tafluprost-containing-preservative or preservative-free-Tafluprost groups after switching.

**Conclusion:**

After switching from preservative Latanoprost to Tafluprost containing-preservative or preservative-free Tafluprost, corneal epithelial barrier function was improved while the intraocular pressure reduction was retained.

## 1. Introduction

Currently, ophthalmic antiglaucoma agents with various mechanisms of action are available, and the range of treatment options for glaucoma has increased. With long-term pharmacotherapy for glaucoma, attention must be paid to side effects in addition to the principle objective of reduction of intraocular pressure (IOP). The well-known characteristic side effects of prostaglandins (PG), the most commonly used drug used to treat glaucoma, include iris pigmentation [1], eyelid pigmentation [2], eyelash extension [2], and deepening of the upper eyelid sulcus [3]. The side effects that may commonly occur, not only with PG-related drugs, but also with other ophthalmic antiglaucoma agents, include ocular surface diseases (OSDs) such as tear reduction and superficial punctate keratopathy (SPK) [4]. In addition to the ophthalmic antiglaucoma agent itself, the effects of preservatives have been indicated as a causative factor of OSD associated with ophthalmic antiglaucoma agent administration [5]. Benzalkonium chloride (BAK) is used as a preservative in several ophthalmic antiglaucoma agents. There are numerous reports on the effects of BAK on corneal epithelial cells in vitro [6–12]. It has been indicated that BAK also causes dry eye in vivo [13]. In addition, BAK-related tear film instability, loss of goblet cells, and disruption of the corneal epithelium barrier have also been reported [14]. In order to reduce the effects of BAK, it may be necessary to decrease its concentration, use a preservative other than BAK [15, 16], or use an ophthalmic antiglaucoma agent that does not contain a preservative. It is well known that the BAK concentration of PG-related TAPROS^®^ ophthalmic solution 0.0015% (BAK-preserved Tafluprost) is low (0.001%) in comparison with that (0.02%) of the current PG-related drug Xalatan^®^ eye drop 0.005% (BAK-preserved Latanoprost). Furthermore, in Japan, not only BAK but also TAPROS Mini ophthalmic solution 0.0015% (preservative-free Tafluprost), available as single-use sterile disposable containers without other preservatives, could be used, thereby making it possible to treat glaucoma without the effects of preservatives.

Therefore, we examined changes in IOP and ocular surface following a switch to BAK-preserved Tafluprost or preservative-free Tafluprost in patients who were using BAK-preservative Latanoprost.

## 2. Methods

### 2.1. Patients

All the procedures were carried out in accordance with the ethical standards laid down by the committee responsible for supervising human experimentation and according to the principles of the Declaration of Helsinki, as revised in 2013. The study was performed with the approval of the Ethical Committee of St. Marianna University School of Medicine (ethical committee approval number: 2912). All the patients provided written informed consent for participation in the study.

This was a 3-month prospective, observer masked study. Patients with early-to-moderate primary open-angle glaucoma, who were treated with BAK-preserved Latanoprost monotherapy for six or more months, were enrolled in the trial. Age between 20 and 80 years was an additional eligibility criterion for the enrollment. The diagnosis of primary open-angle glaucoma was made by a glaucoma expert (NT) based on the Japan Glaucoma Society Guidelines for Glaucoma (3rd Edition) [17] criteria. The patients of the study had to be capable of understanding study instructions and complying with study medication usage and be willing to attend all follow-up visits.

### 2.2. Exclusion Criteria

We excluded patients who met the following criteria:

1. Contact lens use
−4 diopter or greater astigmatism
3. Underlying diseases that may cause corneal disorder
4. Use of an ophthalmic preparation that may induce corneal disorder
5. Severe dry eye
6. A history of intraocular, conventional, or laser surgery in the eye under study (within 6 months prior to enrollment).

### 2.3. Procedures

After providing informed consent, the eligible participants underwent an IOP assessment and an ocular surface evaluation (as described in a later section) after treatment with BAK-preserved Latanoprost. The eligible participants were randomized into two groups as follows: patients who switched from BAK-preserved Latanoprost to BAK-preserved Tafluprost (Tafluprost group), and those who switched from BAK-preserved Latanoprost to preservative-free Tafluprost (PF-Tafluprost group). Subjects were randomized by block randomization. The IOP assessments and ocular surface evaluations were carried out at one month and three months after the eye-drop switch. The IOP measurements were performed by the same masked investigators using Goldmann applanation tonometry, from 9:00 to 11:00 am, in a sitting position.

### 2.4. Ocular Surface Evaluations

We evaluated the ocular surface using the following tests:

1. To evaluate corneal epithelial barrier function, a slit-lamp fluorophotometer (FL-500^®^, Kowa, Tokyo, Japan) was used, for the anterior eye. According to the method of Yokoi and Kinoshita [18], background fluorescence intensity of the central cornea was measured. Fluorescein sodium solution (0.5%) dissolved in BSS PLUS^®^ (3 *μ*L, Alcon, Fort Worth, TX) was applied, avoiding contact, to the lower conjunctival sac, using a micropipette. Eyes were washed with BSS PLUS (20 mL) 10 min after the application. Fluorescein uptake was measured 30 min after the application using the same protocol used for the baseline measurements. The background was subtracted, and the fluorescein uptake concentration was calculated based on a standard curve provided as a built-in function of the FL-500. The data were expressed as ng/mL (normal value: 28 ± 16 ng/mL). For example, values over 50 ng/mL indicated corneal epithelial barrier dysfunction [19].
2. Slit-lamp microscopy was used to measure the corneal status and tear film breakup time (TBUT) [20]. To measure TBUT, fluorescein sodium was applied to the eye, and the patient was instructed to blink several times to facilitate uniform distribution. The time until the occurrence of dry spots in the cornea of the open eye was measured thrice, and the mean of the measurements was used for analysis.
3. The severity of SPK was evaluated using area-density (AD) classification [21], which is a measure of the extent of the lesion (area) and the density of the spotted stain.

### 2.5. Statistics

Data were analyzed using IBM^®^ SPSS^®^ Statistics 21 (IBM Corporation, Poughkeepsie, NY). The data from each examination was analyzed using a paired *t*-test, and a *p* value less than 0.05 was considered statistically significant.

## 3. Results

### 3.1. Patients

Forty patients (33 women and 7 men) with open-angle glaucoma who were treated with BAK-preserved Latanoprost monotherapy completed the study. The mean (±SD) age of participants was 62.8 ± 13.1 years. The study patients were randomized to either BAK-preserved Tafluprost (Tafluprost group: 5 men, 15 women; mean age 60.5 ± 10.9 years) monotherapy dosed in the evening (22:00) or preservative-free Tafluprost (PF-Tafluprost group: 2 men, 18 women; mean age 63.5 ± 10.9 years) monotherapy dosed in the evening (22:00) by block randomization.  Table 1 shows baseline characteristics of both groups.

### 3.2. IOP

**Figure 1:**
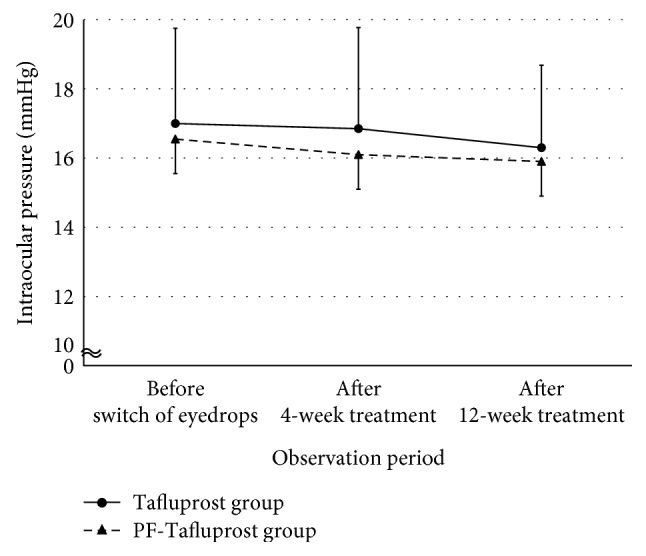
Changes in intraocular pressure in the Tafluprost and PF-Tafluprost groups. Both groups maintained reduced intraocular pressure without significant difference during follow-up.

We assessed changes in the IOP of patients on BAK-preserved Tafluprost or preservative-free Tafluprost treatment (Figure 1). The Tafluprost group retained the reduced IOP without significant differences from the baseline during follow-ups (17.0 ± 2.8 mmHg at baseline; 16.9 ± 2.9 mmHg after 4 weeks; 16.3 ± 2.4 mmHg after 12 weeks); similarly, no change in IOP was observed in the PF-Tafluprost group (16.6 ± 2.5 mmHg at baseline; 16.1 ± 1.8 mmHg after 4 weeks; 15.9 ± 2.3 mmHg after 12 weeks). Importantly, both groups retained the reduced IOP without significant differences during the follow-up period.

### 3.3. Ocular Surface Assessment

**Figure 2:**
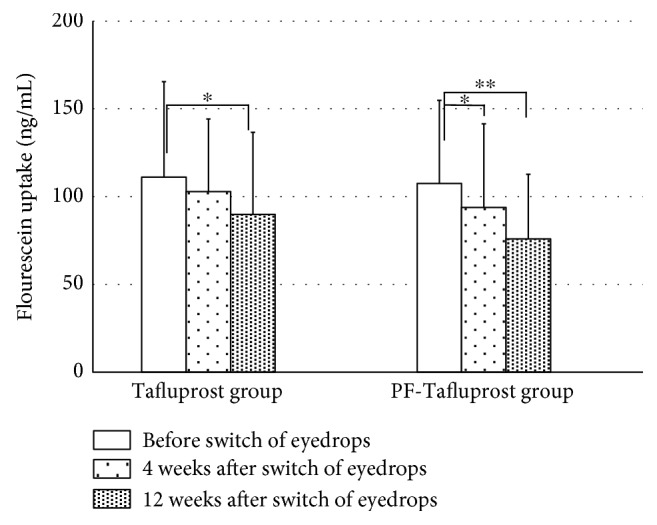
Changes in fluorescein uptake in the patients with glaucoma. Significant decrease in fluorescein uptake was observed in the Tafluprost group after 12 weeks and PF-Tafluprost group after 4 and 12 weeks compared with baseline. ^∗^ paired *t*-test, *p* < 0.05, ^∗∗^ paired *t*-test, *p* < 0.01.

We assessed the corneal epithelial barrier function in patients receiving BAK-preserved Tafluprost or preservative-free Tafluprost (Figure 2). Notably, substantial increases in fluorescein uptake were observed in both the groups at baseline, since both groups received BAK-preserved Latanoprost, indicating corneal epithelial barrier dysfunction induced by BAK-preserved Latanoprost. No significant difference in fluorescein uptake was observed in the Tafluprost group at 4 weeks (111.1 ± 54.3 ng/mL at baseline; 104.5 ± 40.9 ng/mL after 4 weeks [*p* = 0.488]). However, a significant decrease in fluorescein uptake was observed in the Tafluprost group (90.6 ± 45.8 ng/mL [*p* = 0.042]) after 12 weeks. On the other hand, a significant decrease in fluorescein uptake was observed in the PF-Tafluprost group after 4 and 12 weeks (107.5 ± 47.3 ng/mL at baseline; 93.9 ± 47.6 ng/mL after 4 weeks [*p* = 0.033]; and 91.5 ± 37.9 ng/mL after 8 weeks [*p* = 0.017]).

**Figure 3:**
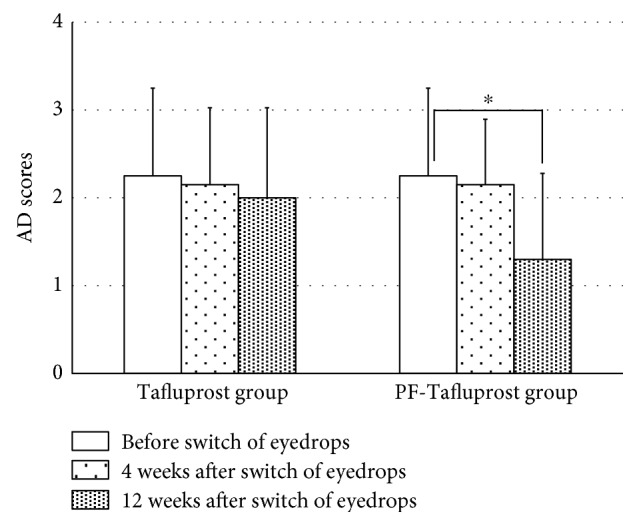
Changes in AD scores in the Tafluprost and PF-Tafluprost groups. No significant difference was observed in the Tafluprost group. No significant improvement in keratopathy was observed in the PF-Tafluprost group at 4 weeks, but significant improvements were observed at 12 weeks. ^∗^ paired *t*-test, *p* < 0.05.

We assessed SPK in patients using the AD classification (Figure 3). There was no statistical difference between the Tafluprost and PF-Tafluprost groups at baseline (2.3 ± 1.0 points and 2.3 ± 0.9 points, resp., *p* = 1.00). No significant difference was observed in the Tafluprost group (2.3 ± 1.0 points at baseline; 2.2 ± 0.9 points after 4 weeks, *p* = 0.16; and 2.0 ± 1.0 points after 12 weeks, *p* = 0.14). Although no significant improvement in keratopathy was observed in the PF-Tafluprost group at 4 weeks (2.2 ± 0.7 points after 4 weeks, *p* = 0.16), significant improvements were observed at 12 weeks (1.3 ± 1.0 points after 12 weeks, *p* < 0.001).

**Figure 4:**
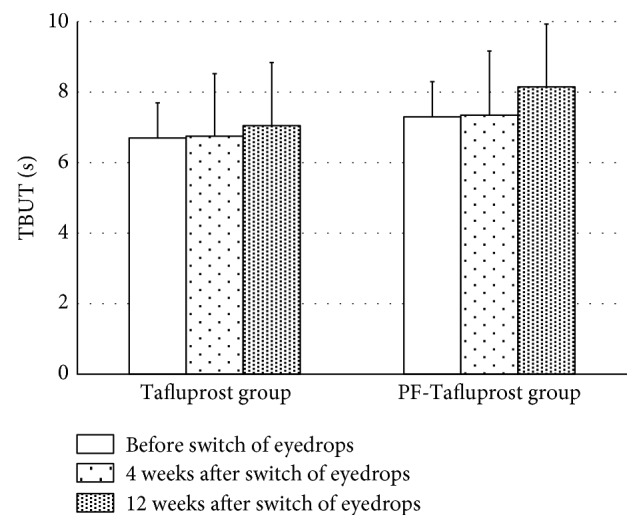
Changes in TBUT in the Tafluprost and PF-Tafluprost groups. No significant differences were observed in either of the groups.

We analyzed TBUT in patients receiving BAK-preserved Tafluprost or preservative-free Tafluprost (Figure 4). There was no statistical difference between the Tafluprost and PF-Tafluprost groups at baseline (6.7 ± 1.8 s and 7.3 ± 2.1 s, resp., *p* = 0.340). No significant differences were observed in either of the groups (Tafluprost group: 6.7 ± 1.8 s at baseline, 6.8 ± 1.8 s after 4 weeks, *p* = 0.58, and 7.1 ± 1.8 s after 12 weeks, *p* = 0.149; PF-Tafluprost group: 7.3 ± 2.1 s at baseline, 7.4 ± 1.8 s after 4 weeks, *p* = 0.80, and 8.2 ± 1.8 s after 12 weeks, *p* = 0.06).

## 4. Discussion

We examined changes in IOP after switching to BAK-preserved Tafluprost or preservative-free Tafluprost in patients who were receiving BAK-preserved Latanoprost; our results revealed no significant differences in either group after switching from BAK-preserved Latanoprost. The IOP-reducing effect of BAK-preserved Tafluprost has been reported to be equivalent to that of BAK-preserved Latanoprost [22, 23]. It has also been reported that in the case of a switch from BAK-preserved Latanoprost to preservative-free Tafluprost, no significant differences in IOP were noted [24, 25]. A comparison of the IOP-reducing effects of BAK-preserved Tafluprost and preservative-free Tafluprost revealed no significant differences between the groups [26]. The results of the present study revealed no significant differences after the switch from BAK-preserved Latanoprost to BAK-preserved Tafluprost or preservative-free Tafluprost; it is important to note that IOP was maintained even after the switch.

We focused on the ocular surface conditions after the switch from Latanoprost to Tafluprost. The fluorophotometric assessments performed in the present study involved a method to numerically assess the corneal epithelial barrier function; the results of the analysis signify that when the fluorescein uptake concentration is high, corneal epithelial barrier function decreases. In the present study, fluorescein uptake concentration with the use of BAK-preserved Latanoprost was clearly higher than the normal values in both the groups, suggesting that corneal epithelial barrier function is affected by long-term use of BAK-preserved Latanoprost. Ishibashi et al. reported that when an ophthalmic solution of BAK-preserved Latanoprost was administered for 30 days, fluorescein uptake concentration was 54.6 ± 7.5 ng/mL and there was an insignificant effect of BAK-preserved Latanoprost on corneal epithelial barrier function [27]. In contrast, our results revealed that after ophthalmic administration of BAK-preserved Latanoprost, the fluorescein uptake concentration was clearly higher, suggesting the unfavorable effects of BAK-preserved Latanoprost on corneal epithelial barrier function. This may be due to the higher age of subjects and the long treatment period of BAK-preserved Latanoprost in the present study. It is interesting to note that corneal epithelial barrier dysfunction was ameliorated after switching from long-term administration of BAK-preserved Latanoprost to Tafluprost containing-preservative or preservative-free Tafluprost. In addition to the ophthalmic antiglaucoma agent itself, the effects of the preservative, especially BAK, have been indicated to be a factor influencing corneal epithelial barrier function [6–14, 19, 28]. The BAK concentration of the BAK-preserved Latanoprost in the present study (0.02%) was twenty times higher than that of the BAK-preserved Tafluprost (0.001%); this suggests that there may be a difference in corneal epithelial barrier function due to long-term use of an ophthalmic solution. Nakagawa et al. examined the effects of BAK-preserved Latanoprost and BAK-preserved Tafluprost on human corneal epithelial function and indicated that Tafluprost had less influence than Latanoprost [29]. Our results show that when BAK-preserved Latanoprost was changed to BAK-preserved Tafluprost, corneal epithelial barrier function did not recover at four weeks after the switch in healthy persons, but by switching from BAK-preserved Latanoprost to preservative-free Tafluprost, corneal epithelial barrier function began to recover at four weeks, and the recovery was maintained at 12 weeks after the switch. In the case of SPK, when the switch was made from BAK-preserved Latanoprost to BAK-preserved Tafluprost, no significant differences in AD scores at 12 weeks following the switch were noted, but AD scores were significantly lower at 12 weeks following the switch from BAK-preserved Latanoprost to preservative-free Tafluprost. SPK was found to lead to decreased corneal epithelial barrier function. It appears that by decreasing the effects of BAK, corneal epithelial barrier function and SPK were ameliorated. TBUT is a widely used noninvasive method that can evaluate the stability of tear film [20]. Terai et al. examined the changes in the ocular surfaces of healthy persons following the administration of various ophthalmic antiglaucoma agents and reported a shortening of TBUT following administration of BAK-preserved Latanoprost and 0.02% BAK solution. The results of the present study revealed that in both the Tafluprost and PF-Tafluprost groups, no significant differences with respect to TBUT were noted with switching, but both groups showed a trend toward prolonged TBUT [30]. Based on these results, reducing the effects of BAK may lead to stability of the tear film, but further long-term follow-up observations are necessary.

There are several limitations in the present study. First, we did not provide the washout period because it may be accompanied by an elevation of IOP. Second, high percentage of women may affect the present results. Third, we did not evaluate the symptoms between before and after switching the eye drop.

In conclusion, these findings suggest that switching to BAK-preserved Tafluprost or preservative-free Tafluprost, in patients with an onset of OSD owing to the use of an ophthalmic antiglaucoma agent containing BAK-preserved Latanoprost, may be an effective option.

## Figures and Tables

**Table 1:** Baseline characteristics.

	Tafluprost group (*n* = 20)	PF-Tafluprost group (*n* = 20)	*p* values
Gender (male/female)	5/15	2/18	
Age (years)	60.5 ± 0.9	63.5 ± 10.9	0.397
Intraocular pressure (mmHg)	16.6 ± 2.3	16.2 ± 2.3	0.592
Fluorescein uptake (ng/mL)	111.1 ± 54.3	107.5 ± 47.3	0.824
TBUT (s)	6.7 ± 1.8	7.3 ± 2.1	0.340
AD scores	2.3 ± 1.0	2.3 ± 0.9	1.00

Results are presented as mean ± standard deviation.
